# Th1 Cells Alter the Inflammatory Signature of IL-6 by Channeling STAT Transcription Factors to *Alu*-like Retroelements

**DOI:** 10.4049/jimmunol.2300114

**Published:** 2023-06-05

**Authors:** David Millrine, Ana Cardus Figueras, Javier Uceda Fernandez, Robert Andrews, Barbara Szomolay, Benjamin C. Cossins, Christopher M. Rice, Jasmine Li, Victoria J. Tyrrell, Louise McLeod, Peter Holmans, Valerie B. O’Donnell, Philip R. Taylor, Stephen J. Turner, Brendan J. Jenkins, Gareth W. Jones, Nicholas Topley, Nigel M. Williams, Simon A. Jones

**Affiliations:** * Division of Infection and Immunity, School of Medicine, Cardiff University, Cardiff, Wales, United Kingdom; † Systems Immunity University Research Institute, Cardiff University, Cardiff, Wales, United Kingdom; ‡ Medical Research Council Protein Phosphorylation and Ubiquitylation Unit, School of Life Sciences, University of Dundee, Dundee, United Kingdom; § School of Cellular and Molecular Medicine, University of Bristol, Bristol, United Kingdom; ¶ Department of Microbiology, Biomedicine Discovery Institute, Monash University, Clayton, Victoria, Australia; ‖ Centre for Innate Immunity and Infectious Diseases, Hudson Institute of Medical Research, Clayton, Victoria, Australia; # Department of Molecular Translational Science, Faculty of Medicine, Nursing and Health Sciences, Monash University, Clayton, Victoria, Australia; ** Division of Psychological Medicine and Clinical Neuroscience, School of Medicine, Cardiff University, Cardiff, Wales, United Kingdom; †† UK Dementia Research Institute at Cardiff, Cardiff University, Cardiff, Wales, United Kingdom

## Abstract

Cytokines that signal via STAT1 and STAT3 transcription factors instruct decisions affecting tissue homeostasis, antimicrobial host defense, and inflammation-induced tissue injury. To understand the coordination of these activities, we applied RNA sequencing, chromatin immunoprecipitation sequencing, and assay for transposase-accessible chromatin with high-throughput sequencing to identify the transcriptional output of STAT1 and STAT3 in peritoneal tissues from mice during acute resolving inflammation and inflammation primed to drive fibrosis. Bioinformatics focused on the transcriptional signature of the immunomodulatory cytokine IL-6 in both settings and examined how profibrotic IFN-γ–secreting CD4^+^ T cells altered the interpretation of STAT1 and STAT3 cytokine cues. In resolving inflammation, STAT1 and STAT3 cooperated to drive stromal gene expression affecting antimicrobial immunity and tissue homeostasis. The introduction of IFN-γ–secreting CD4^+^ T cells altered this transcriptional program and channeled STAT1 and STAT3 to a previously latent IFN-γ activation site motif in *Alu*-like elements. STAT1 and STAT3 binding to this conserved sequence revealed evidence of reciprocal cross-regulation and gene signatures relevant to pathophysiology. Thus, we propose that effector T cells retune the transcriptional output of IL-6 by shaping a regulatory interplay between STAT1 and STAT3 in inflammation.

## Key Points

- Th1 cells alter the transcriptional output of IL-6 during acute inflammation.
- IL-6 and IFN-γ activities combine to steer tissue responses to inflammation.
- STAT1 and STAT3 work in partnership to regulate immune outcomes.

## Introduction

Cytokines are essential for development, tissue homeostasis, and the regulation of inflammation (1). The intracellular signaling pathways controlling these activities are intrinsically linked, and aberrant host defense compromises tissue integrity and physiological function. Patients treated with anti-cytokine therapies often provide evidence of this relationship (2). For example, IL-6 inhibition is less effective in diseases where IL-6 maintains tissue homeostasis and barrier immunity (3–10). How IL-6 operating through a single receptor signaling cassette coordinates the maintenance of tissue physiology and the transition to pathophysiology is unknown.

IL-6 regulates cellular responses via receptor activation of Jaks and members of the STAT family (3, 6). Although IL-6 employs other signaling intermediates, it instructs cell decisions primarily through STAT1 and STAT3 transcription factors (11–17). These proteins share a complex regulatory interplay, and gene ablation studies show that STAT1 and STAT3 often counteract each other or engage shared enhancers (3, 18–20). These interactions retune the interpretation of cytokine cues, instructing alternate patterns of gene regulation (14, 16, 20–26).

Resident tissue cells respond to immune challenges by steering decision-making processes affecting the disease outcome (1, 27–29). These activities rely on cytokine networks that promote communication between stromal tissue and infiltrating leukocytes (29). In bacterial peritonitis, IL-6 controls antimicrobial immunity and the resolution of inflammation by steering the transition from innate to adaptive immunity (30–35). This process requires IL-6R shedding from infiltrating neutrophils, which promotes IL-6 *trans*-signaling and STAT3-driven outcomes that instruct the tissue response to peritonitis (33, 34, 36–38). Repeated episodes of acute inflammation disrupt this regulatory mechanism by promoting the expansion of profibrotic IFN-γ–secreting Th1 cells, which enhance tissue injury through the activation of STAT1 signaling (22). These findings suggest a regulatory network involving IL-6 and IFN-γ and raise the possibility that the shift in the STAT1 signaling dynamic may alter the transcriptional output of STAT3 in acute inflammation.

To understand how the effector properties of Th1 cells impact the contribution of STAT1 and STAT3 in acute resolving peritonitis, we have applied next-generation sequencing methods to examine the stromal response to inflammation. Our analysis shows that Th1 cells alter the transcriptional output of IL-6 by redirecting STAT transcription factors to a IFN-γ activation site (GAS)–like motif in *Alu*-like retroelements. These results offer an explanation of how effector lymphocytes shape the stromal response to inflammation by modifying the interpretation of cytokine cues.

## Materials and Methods

### Animals

All procedures were performed under UK Home Office project license P05D6A456. Inbred wild-type (wt) C57BL/6 male mice were purchased from Charles River U.K. IL-6–deficient (*Il6*^−/−^) mice (39) were bred under approved UK Home Office guidelines in Cardiff University. The *gp130*^Y757F:Y757F^ and *gp130*^Y757F:Y757F^:*Stat3*^+/−^ mice have been previously described (40). Experiments were approved by the Animal Ethics Committee and included genetically matched *gp130*^+/+^ littermate controls. All experiments were performed using age-matched 8- to 12-wk-old mice.

### *Staphylococcus epidermidis*–induced peritoneal inflammation

A lyophilized cell-free supernatant prepared from *S. epidermidis* (SES), whose activity had been standardized using an in vitro cell-based CXCL8 bioassay, was used to induce acute peritoneal inflammation (33). Mice were i.p. administered with a defined dose of SES (500 μl). Soluble gp130Fc was added i.p. to wt mice as indicated. At 3 and 6 h after inflammatory challenge, mice were sacrificed and the peritoneal cavity was lavaged with 2 ml of ice-cold PBS. The peritoneal membrane was harvested at the same time points.

### Transfer of Th1 cells

To replicate events promoting peritoneal fibrosis, some mice receiving SES were simultaneously administered with either naive CD4^+^ T cells or CD4^+^ T cells conditioned ex vivo toward a Th1 phenotype. Briefly, splenic naive CD4^+^ T cells (CD4^+^CD25^−^CD44^lo^CD62L^hi^) were flow sorted and place in coated plates with anti-CD3e (145-2C11) and 5 μg/ml soluble anti-CD28 (37.51) Abs. Cells were cultured for 4 d in the presence of 20 ng/ml murine rIL-12 (R&D Systems, 419-ML) or conditioned medium from SES–activated peritoneal monocytic cells (22). The proportion of IFN-γ–secreting CD4^+^ T cells (Th1 cells) was determined by intracellular flow cytometry using Abs against CD4 (RM4-5), IFN-γ (XMG1.2), and IL-4 (11B11) (Supplemental Fig. 1). The reader is directed elsewhere for further information on the effector characteristics of these expanded cells (22). T cells were washed in ice-cold PBS, resuspended in a 500-μl aliquot of PBS-reconstituted SES, and administered i.p. to mice at a cell concentration of 5–10 × 10^5^ CD4^+^ T cells. This cell number reflected the proportion of Th1 cells recruited to the peritoneal cavity under acute SES challenge (22, 32, 35). To control for the addition of Th1 cells, a separate group of mice were administered with an equivalent number of sorted naive (Th0) CD4^+^ T cells (Supplemental Fig. 1C).

### Fluorescent labeling of bacteria

An inoculum of *S. epidermidis* ATCC 12228 (5 × 10^8^ CFU/mouse) was prepared from log-phase cultures under sterile conditions. The suspension was centrifuged and bacteria were labeled for 20 min at 37°C in prewarmed PBS containing Cell Trace Far Red (CT-FR) (Life Technologies) (1 or 8 μM for ex vivo and in vivo experiments, respectively). For ex vivo experiments, bacteria were serum-opsonized whereas for in vivo experiments they were centrifuged and washed three times in PBS before resuspension in sterile PBS.

### Ex vivo neutrophil effector function assay

Whole blood was collected by cardiac puncture into tubes containing 5 mM EDTA. Samples were diluted 1:10 and washed three times in ice cold PBS. Cells were resuspended in serum-free RPMI 1640 containing 5 µM 3′-(*p*-aminophenyl)fluorescein (APF) and incubated for 30 min at 37°C. APF-loaded cells were split into 100-µl aliquots and cultured at 37°C with an equal volume of prewarmed, opsonized CT-FR (1 µM)–labeled *S. epidermidis*. Cells were incubated for set time intervals (0–30 min) and transferred to an iced water bath prior to preparation for flow cytometry using a Beckman Coulter CyAn-ADP flow cytometer.

### In vivo neutrophil effector function assay

Mice were i.p. administered with CT-FR (8 µM)–labeled *S. epidermidis*. An independent group of *Il6*^−/−^ mice received a dose of CT-FR–labeled *S. epidermidis* and the IL-6–soluble IL-6R (sIL-6R) chimeric fusion protein HDS (50 ng/ml). Six hours after bacterial challenge, the peritoneal cavity was lavaged with 2 ml of RPMI 1640 containing 5 µM APF. Neutrophil phagocytosis and respiratory burst activity were examined by flow cytometry using a Beckman Coulter CyAn-ADP flow cytometer and analyzed using Summit Software v4.3 (Beckman Coulter) or FlowJo 10 (Tree Star).

### Imaging flow cytometry analysis

Lavaged neutrophils were immune-stained for Ly6G (1A8), resuspended in 100 µl of sterile PBS, and events (>8000 events/sample) were acquired at a low imaging rate and 60× amplification using the Amnis ImageStream^X^ Mark II imaging flow cytometer (Amnis). Neutrophils were gated according to Ly6G staining. Phagocytic activity was expressed as a phagocytic index reflecting the number of bacteria ingested by an individual Ly6G^+^ neutrophil during the incubation. The “spot counting” function in ImageStream software IDEAS (Amnis) allowed determination of the phagocytic index based on the distribution of CT-FR bacterial staining. The efficiency of phagocytic uptake was quantified by examining cells displaying either a low (one to two counts) or a high number (three counts or more) of ingested bacteria.

### Immunoblotting of peritoneal tissues

Protein was extracted from frozen peritoneal biopsies using ice-cold lysis buffer. Samples were precleared of cellular debris before separation by SDS-PAGE and immunoblotting with specific Abs against STAT1, STAT3, and tyrosine-phosphorylated STAT1 (pY-STAT1) and STAT3 (pY-STAT3) (40). Immunolabeled proteins were detected by ECL (Amersham Biosciences) on an Odyssey infrared imaging system (LI-COR Biosciences, Lincoln, NB) using appropriate secondary Abs as per the manufacturer’s instructions.

### RNA sequencing

Peritoneal membrane sections (80 mg of tissue) were dissociated in 1 ml of Buffer RLT (Qiagen) supplemented with 2-ME (1:100 v:v) using a handheld electric homogenizer (Benchmark Scientific). Lysate was diluted 1:3 in distilled water and digested in 0.2 mg/ml proteinase K (Invitrogen, 25530049) for 10 min at 55°C. Lysate was cleared and RNA was precipitated in 70% ethanol. Total RNA was extracted using the RNeasy mini kit (Qiagen) following the manufacturer’s instructions. Two to 4 mg of mRNA was processed to generate the libraries. Cytoplasmic RNA, mitochondrial RNA, and rRNA were depleted using the RiboMinus transcriptome isolation kit (Ambion, K155001). Libraries were prepared using the RNA sequencing (RNA-seq) kit v2 (Life Technologies, 4475936) and sequenced on an ion torrent (Thermo Fisher Scientific).

### Chromatin immunoprecipitation sequencing

Excised peritoneal membranes were immediately frozen in liquid nitrogen and stored at −80°C until use. Tissues were diced and ground to a fine powder with intermittent addition of liquid nitrogen. Genomic DNA was extracted, crosslinked, and fragmented by sonication prior to treatment with 2 μg/ml anti-STAT1 (Santa Cruz Biotechnology, sc-592), anti-STAT3 (Santa Cruz Biotechnology, sc-482), anti-P300 (Millipore, 05-257), or isotype control Abs. Immunoprecipitation was conducted overnight at 4°C under continuous gentle agitation. Ag–Ab complexes were captured using protein A/G magnetic beads, washed, and DNA fragments were liberated following treatment with proteinase K and extraction with phenol/chloroform. Biological repeats from three independent tissue extracts were pooled and concentrated before library preparation and sequencing. Chromatin immunoprecipitation (ChIP) libraries were prepared according to the manufacturer’s instructions (Illumina TruSeq DNA ChIP kit, RS-122-2001). Size selection (200–400 bp) was determined using a BluePippin (Sage Science) system employing 2% agarose cartridges (Sage Science, BDF2003). Appropriate library size distribution was confirmed on an Agilent 2100 Bioanalyzer and quantified (Qubit; Invitrogen) prior to sequencing (Illumina HiSeq 4000).

### Assay for transposase-accessible chromatin with high-throughput sequencing

Excised peritoneal membranes were immediately frozen in liquid nitrogen and stored at −80°C until use. Tissues were diced and ground to a fine powder with intermittent addition of liquid nitrogen. Omni–assay for transposase-accessible chromatin with high-throughput sequencing (ATAC-seq) was performed as described (41). Briefly, 100,000 nuclei per sample were isolated using an iodixanol gradient, and ATAC-seq was performed according to the original protocol (42) using a Nextera DNA sample preparation kit (Illumina, FC-121-1030). After amplification, library DNA was isolated (Qiagen MinElute kit), size selected, and sequenced (Illumina HiSeq 4000).

### Quantification and statistical analysis

No randomization and no blinding were used for the animal experiments. Whenever possible, the investigator was partially blinded for assessing the outcome. All data were analyzed using Prism 8 (GraphPad Software, La Jolla, CA). Information on the types of statistical methods used, the sample size, and the number of repetitions are listed in the figure legends.

### RNA-seq data analysis

Raw fastq files were mapped using Torrent Suite to the *mm10* reference genome and counts were assigned to transcripts using featureCounts (43) with the GRCm38.84 Ensembl gene build GTF. Differential gene expression analyses used the DESeq2 package (44). Genes below the significance or log fold change thresholds (padj < 0.05; log2FC > 1.75) were omitted from the analysis. Differentially regulated genes were uploaded into Ingenuity Pathway Analysis (Qiagen) for functional analysis.

### ChIP sequencing data analysis

Between 40 and 70 million reads were obtained for each sample. These were trimmed with Trim Galore (https://www.bioinformatics.babraham.ac.uk/projects/trim_galore/) and assessed for quality using FastQC (https://www.bioinformatics.babraham.ac.uk/projects/fastqc/). Reads were mapped to the mouse *mm10* reference genome using bwa (45). Peaks were called using macs2 (46), using the BAMPE model (*p*_adj_ < 0.05).

### ATAC-seq analysis

Paired-end reads were processed with Trim Galore and assessed for quality using FastQC (https://www.bioinformatics.babraham.ac.uk/projects/fastqc/) prior to mapping to the mouse *mm10* reference genome (45). Peaks were called using macs2 and the BAMPE model (*p*_adj_ < 0.05) (46). Differential open-region analysis used DiffBind in Bioconductor (http://bioconductor.org/packages/release/bioc/vignettes/DiffBind/inst/doc/DiffBind.pdf).

### FANTOM5 enhancer analysis

Sequences annotated as enhancers by the FANTOM5 consortium were downloaded from Slidebase (250-bp pad) (http://slidebase.binf.ku.dk). Motif occurrences (*n* = 8423, *p* < 0.00001) in these sequences were identified using the FIMO algorithm (MEME-ChIP suite; http://meme-suite.org/tools/fimo). Genes in proximity to the identified sequences (defined as 2 kb upstream or downstream) were mapped to the Ensembl GRCh38 (hg38) build and visualized using the ClueGO Cytoscape plugin. Genome-wide motif occurrences were visualized with the RIdeogram R package (https://cran.r-project.org/web/packages/RIdeogram/vignettes/RIdeogram.html).

### Motif identification

Sequences under ChIP peaks (*q* < 0.05) were obtained using the bedtools getfasta command against the ensemble *mm10* genome build. Sequence fasta files were uploaded to MEME-ChIP (http://meme-suite.org/tools/meme-chip) and searched against the murine HOCOMOCO (v11 CORE) and eukaryote DNA databases. The de novo GAS-like motif was enriched by both CentriMo and MEME algorithms.

### Spaced motif analysis

Analysis was conducted with SpaMo (MEME-ChIP suite) using default parameters. Secondary motifs occur within 150 bp of the user-provided primary motif (GAS-like). All secondary motifs are referenced in the HOCOMOCO (v11 CORE) database. Input sequences, including a 250-bp pad, were derived from the ChIP sequencing (ChIP-seq) data or enhancer annotations in FANTOM5.

### Multiple sequence alignment

Consensus sequences (hidden Markov models) corresponding to murine and human Alu family transposable elements were downloaded from the Dfam database (https://dfam.org/) as follows: mice: B1_mm, B1_mus1, PB1D11, B1_Mur2, B1_Mur4, PB1, B1_Mur1, B1_Mur3, PB1D10, B1_Mus2, B1_F1, PB1D7, PB1D9, B1F2, B1F, B2_Mm1a, B2_Mm1t, B2_Mm2, B3, B3A, B4, B4A, and ID_B1; humans: AluY, AluSc, AluJB, AluJo, AluJr, AluJr4, AluSc5, AluSc8, AluSg, AluSg4, AluSg7, AluSp, AluSq, AluSq10, AluSq2, AluSq4, AluSx, AluSx1, AluSx3, AluSx4, AluSz, AluSz6, AluYa5, AluYa8, AluYb8, AluYb9, AluYc, AluYc3, AluYd8, AluYh9, AluYk11, AluYk12, AluYk4, AluYg6, AluYk3, AluYm1, AluYk2, AluYe6, AluYi6, AluYe5, AluYi6_4d, AluYf1, AluYh3, AluYj4, AluYh7, FAM, FLAMA, FLAMC, and FRAM. Sequences were aligned using MUSCLE (European Bioinformatics Institute) with the default ClustalW output visualized in Jalview.

### Gene set enrichment analysis

A ranked gene list was prepared for each dataset using the differential gene expression analysis Log2FC value. Using the gene set enrichment analysis (GSEA) preranked function, enrichment profiles were generated against the biological processes (C-5; 7573 gene sets) Gene Ontology database. For visualization, GSEA output files were loaded into Cytoscape using the enrichment-map plugin. For small gene sets, ontology enrichments were performed using either the MSigDB overlap tool (Broad Institute) or the Metascape online tool. Network aesthetics (e.g., color, spacing) were modified in Adobe Illustrator. Network statistical thresholds are below *p* < 0.01, *q* < 0.01.

### RepeatMasker overlaps

Alu and L1 sequence coordinates were derived from the RepeatMasker definitions downloaded from the UCSC Table Browser (*mm10*). Repeats overlapping STAT binding were identified using the bedtools intersect algorithm. The absolute number of Alu and L1 sequences overlapping each ChIP dataset was calculated in RStudio and plotted using the circlize package.

### Visualization and annotation

Heatmap visualization in Morpheus (Broad Institute) and pheatmap (R package) using colors defined in the viridis and R Color Brewer packages. Figures were prepared using the ggplot2 R package, GraphPad Prism 8, and arranged in Adobe Illustrator. Heatmap visualisations were constructed in pheatmap and ComplexHeatmap R-packages. IL-6–regulated genes were defined as those meeting the statistical threshold (*Il6*^−/−^ versus wt *p*_adj_ <0.05 with a log_2_ fold change (FC) >1.75 or less than −1.75) in at least one experimental condition. STAT-regulated genes were similarly described as those with ChIP signal under the statistical threshold (*q* < 0.05) in at least one experimental condition.

### Generation of gene sets for MAGMA analysis

Sequences annotated as enhancers by the FANTOM5 consortium were downloaded from SlideBase (http://slidebase.binf.ku.dk/) including a 200-bp pad. To identify motif occurrences, these sequences were entered to the FIMO Web server (MEME-ChIP suite) together with the MEME-formatted motif extracted from our ChIP-seq analyses. Identified gene sets were derived by mapping coordinates to the HG19 reference genome (genes within 2 kb) using PAVIS (https://manticore.niehs.nih.gov/pavis2/). In parallel, FANTOM5 sequences containing Alu sequences were defined by intersecting (bedtools) FANTOM5 coordinates with the RepeatMasker database (UCSC Table Browser). Coordinates were mapped to genes using PAVIS. Randomized control gene sets were derived as follows: 1) FIMO output coordinates were “shuffled” across FANTOM sequences or genome wide (Hg19 reference genome) using the bedtools shuffle feature; and 2) random bed files (8000 × 500-bp sequences) were generated using bedtools random against either the FANTOM5 enhancer sequences or genome wide (hg19). Three gene sets were generated for each control. Hallmark gene sets were downloaded from MSigDB (Broad Institute). Genes mapping to the MHC locus were downloaded from the UCSC Table Browser. This gene list was used to filter MHC locus genes from all gene sets prior to MAGMA analysis (dplyr R package).

### MAGMA

GWAS summary statistics (Hg19) were downloaded using FTP links supplied by the GWAS atlas (https://atlas.ctglab.nl/). After reformatting for compatibility with Magma, summary statistics were mapped to genes using the build 37 gene locations file (NCBI37.3.gene.loc). Gene results files were next generated for each summary statistic. The GWAS atlas was used to reference *n* (number of study participants) for each study. Finally, gene set analysis was performed against gene sets generated as described above. Results files were read into R and relevant columns were extracted using R base functions. For genes, *p* values were extracted from MAGMA output files ending genes.sets.out. For gene sets, *p* values were extracted from output files ending.gsa.out. The *p* values were merged into a single dataframe for correlation analysis (R base function) or filtering (dplyr R package). Heatmaps were generated using the pretty heatmap R package (pheatmap).

### Data and code availability

RNA-seq (https://www.ebi.ac.uk/biostudies/arrayexpress/studies/E-MTAB-10087), ChIP-seq (https://www.ebi.ac.uk/biostudies/arrayexpress/studies/E-MTAB-10758), and ATAC-seq (https://www.ebi.ac.uk/biostudies/arrayexpress/studies/E-MTAB-10739) datasets reported in this article have been deposited in ArrayExpress. All R scripts for performing the main steps of analysis are available from the lead contact.

## Results

### Effector Th1 cells shape stromal responses to peritonitis

**FIGURE 1.**
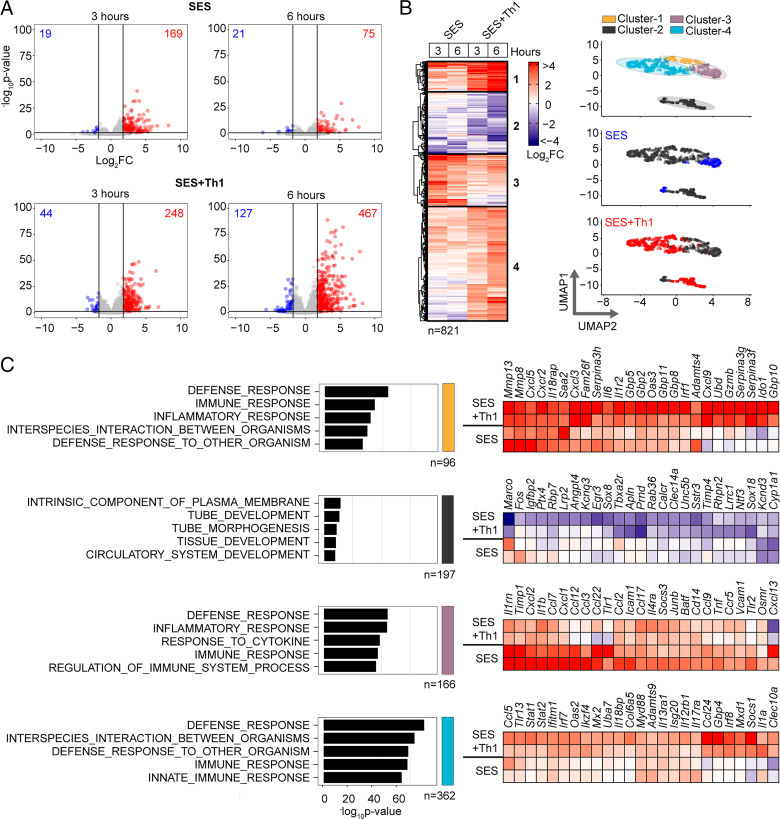
Th1 cells augment the stromal response to acute inflammation. (**A**) RNA-seq was performed on stromal tissues extracted from SES–challenged mice. Volcano plots show differential expression analysis (limma) and statistical thresholding (*p*_adj_ < 0.05, log_2_FC > 1.75) of datasets at 3 and 6 h posttreatment. Mice received i.p. SES alone (SES) or SES in combination with Th1 cells (SES+Th1). Each test condition was compared with untreated mice (SES), or mice receiving SES and naive CD4^+^ T cells. (**B**) K-means clustering (*n* = 4) of data shown in (A) (heatmap). Uniform manifold approximation and projection (UMAP) visualizations show the distribution of gene clusters and their link to SES (blue) or SES+Th1 (red) datasets. (**C**) Gene Ontology (MSigDB) shows the top five biological processes for each cluster (left). Each cluster descriptor is coded to match UMAP clustering colors in (B). Representative examples of genes in each cluster are presented as heatmaps (right).

**FIGURE 2.**
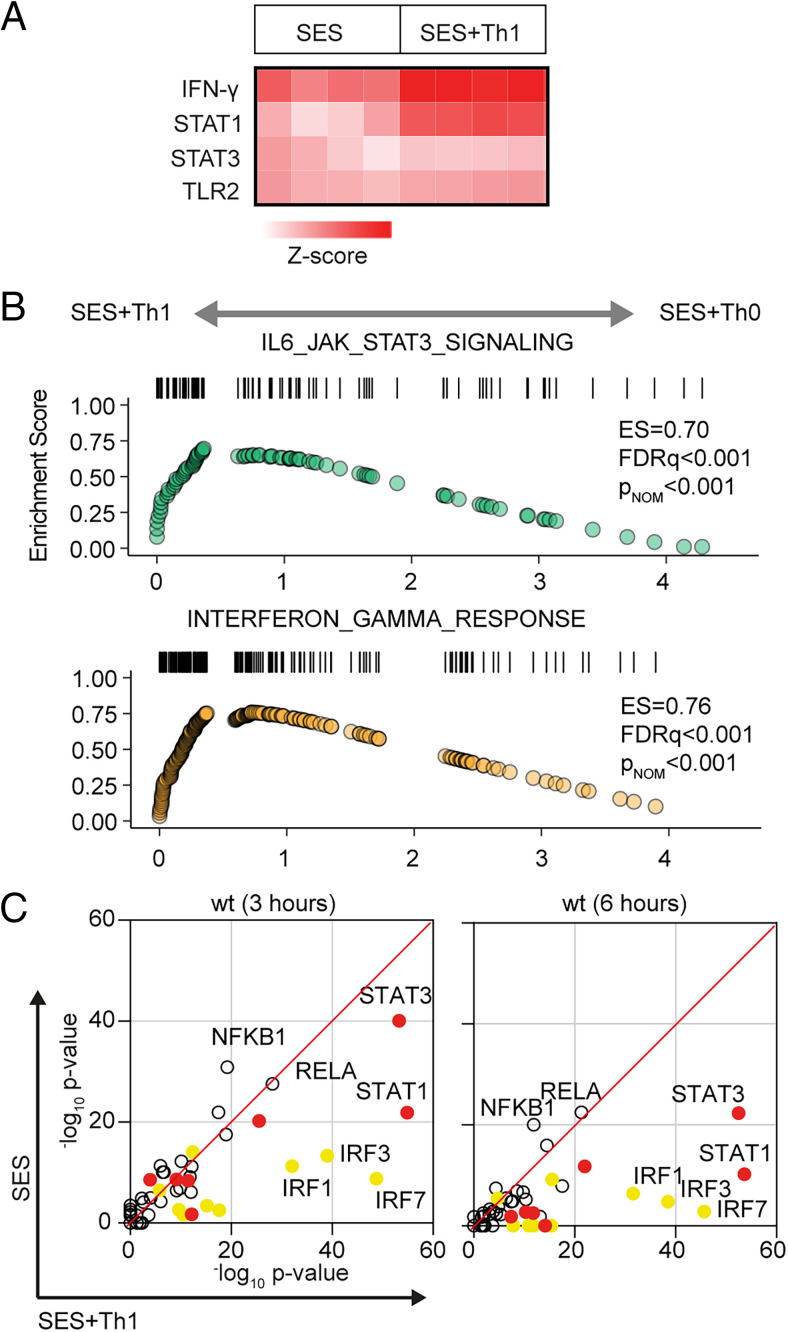
Regulatory signatures of cytokine signaling in acute inflammation. (**A**) Ingenuity Pathway Analysis of biological processes involved in SES–induced inflammation. (**B**) GSEA shows the impact of Th1 cells on Jak–STAT cytokine signaling and IFN-regulated outcomes. A summary of the complete GSEA analysis is provided in Supplemental Fig. 1. (**C**) Ingenuity Pathway Analysis of upstream regulators. Predicted *p* value (Fisher’s exact test) identifies gene signatures characteristic of specific transcription factors linked to TLR (NF-κB, Rel-A are shown), IFN (yellow; IRF family members are shown), and Jak–STAT cytokine signaling (red; STAT1 and STAT3 are displayed).

To understand how profibrotic Th1 cells shape the stromal cell response to acute peritonitis, we challenged mice with an i.p. administration of SES (22, 33, 40). Mice received SES alone or coadministered with CD4^+^ T cells expanded ex vivo into Th1 cells (normalized to 10^6^ IFN-γ–secreting T cells) (22). To control for transcriptional changes induced by Th1 cells (SES+Th1), a separate group of mice received SES and an equivalent number of naive CD4^+^ T cells (Fig. 1, Supplemental Fig. 1). At 3 and 6 h poststimulation, the peritoneum was harvested and prepared for RNA-seq. K-means clustering was restricted to transcripts differentially regulated (log_2_FC > 1.75, *p*_adj_ < 0.05) under each condition (Fig. 1A). Applying statistical tools (silhouette, gap statistics, and elbow), we identified four clusters of gene regulation in mice receiving SES alone or SES+Th1 (Fig. 1B). We identified 821 differentially regulated transcripts whose expression altered in (at least) one time point. SES regulated a total of 225 genes, with the activities of Th1 cells affecting another 673 genes. These included genes controlling vascularization, epithelial morphogenesis, and hyperplasia (e.g., *Angpt4*, *Egr3*, *Igfbp2*, *Ntf3*, *Tbxa2r*). Others displayed involvement in innate sensing pathways (e.g., *Ifitm1*, *Marco*, *Myd88*) and leukocyte infiltration (e.g., *Ccl3*, *Ccl5*, *Ccl2*, *Cxcl1*, *Cxcl10*, *Icam1*, *Vcam1*) (Fig. 1C). The presence of Th1 cells altered gene expression in each cluster, with molecular pathway analysis highlighting an enrichment of transcripts attributed to STAT1 and IFN signaling (Fig. 2A, 2B, Supplemental Fig. 1B). To substantiate these findings, we used the Upstream Regulator tool in Ingenuity Pathway Analysis to predict transcriptional mechanisms accounting for changes in gene expression in each dataset. Consistent with the SES activation of TLR2 (47), this algorithm identified genes controlled by the NF-κB pathway (Fig. 2C). It also identified genes linked with STAT1, STAT3, and several IRF transcription factors (Fig. 2C). Transcripts affiliated with STAT1 and STAT3 showed considerable enrichment in SES+Th1–treated mice, suggesting a potential link between IL-6 and IFN-γ signaling in steering SES–induced outcomes (22, 33, 36, 48).

### Th1 cells modify the properties of IL-6

**FIGURE 3.**
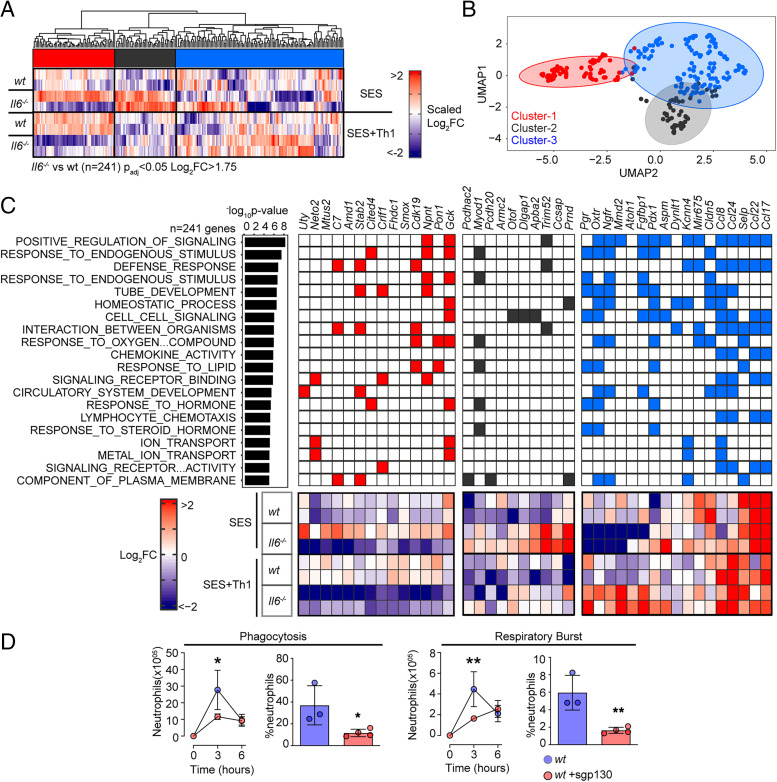
Th1 cells shape the transcriptional output of IL-6. (**A**) Heatmap of IL-6–regulated transcripts identified by differential expression analysis (limma) of wt versus *Il6*^−/−^ mice. Data are arranged by K-means clustering (*n* = 3) of significantly regulated transcripts (cluster 1, red; cluster 2, gray; cluster 3, blue). (**B**) UMAP visualization of all IL-6–regulated transcripts (*p*_adj_ < 0.05, log_2_FC > 1.75). (**C**) Alignment of representative transcripts from each cluster against the top 20 biological processes identified by Gene Ontology enrichment analysis of 241 IL-6–regulated transcripts (MSigDB). (**D**) Flow cytometric analysis of infiltrating neutrophil effector function (see Supplemental Fig. 2). Peritonitis was induced in wt mice by administration of fluorescently labeled *S. epidermidis* (5 × 10^8^ CFU) in the presence of sgp130Fc (250 ng/mouse). Changes in phagocytosis and respiratory burst are shown (mean ± SEM, *n* = 4; *p < 0.05, **p < 0.01).

To explore the link between IL-6 and IFN-γ in SES–induced peritonitis, we used immunodetection methods to quantify changes in their production as a response to SES challenge (Supplemental Fig. 2A). Peritoneal IFN-γ remained below the limit of detection in mice treated with SES alone. However, IFN-γ levels were substantially enhanced by the introduction of Th1 cells (Supplemental Fig. 2A). The presence of Th1 cells also increased IL-6 bioavailability by promoting 3- to 4-fold increases in IL-6 and sIL-6R (Supplemental Fig. 2A). To establish whether Th1 cells alter the transcriptional output of IL-6, we performed RNA-seq on peritoneal tissues from SES–challenged wt and *Il6*^−/−^ mice lacking an ability to signal via classical IL-6R signaling and IL-6 *trans*-signaling. K-means clustering identified 241 significantly altered transcripts (log_2_FC > 1.75, *p*_adj_ < 0.05) impacted by the loss of IL-6 (Fig. 3A, 3B, Supplemental Fig. 2B–D). These included genes required for tissue homeostasis (e.g., *Atoh1*, *Cldn5*, *Fgfbp1*, *Npnt*, *Oxtr*, *Pdx1*, *Pgr*, *Stab2*), host defense (e.g., *Trim52*, *C7*), leukocyte recruitment (e.g., *Ccl8*, *Ccl17*, *Ccl22*, *Ccl24*), and adhesion (e.g., *Selp*, *Adgrb2*) (Fig. 3C). To identify the peritoneal stromal cells responding to IL-6 in SES–induced inflammation, we compared our results against a single-cell RNA-seq dataset (GEO GSM4053741) from mouse omental CD45^−^CD41^−^Ter119^−^CD31^−^PDPN^+/−^ cells (49). This analysis defined roles for peritoneal fibroblasts (including *Ccl11*^+^ *Pdgfra*^+^ and *Matn2*^+^ *Pdgfra*^+^ subsets) and mesothelial cells (including *Ifit*^+^ and *Cxcl13*^+^ mesothelial cells) in shaping IL-6 responses (Supplemental Fig. 2E).

Our initial bioinformatics predictions suggested that IL-6 controls stromal responses affecting innate immunity. To verify this connection, we developed a flow cytometric method to compare the effector properties of circulating and infiltrating Ly6B^hi^Ly6G^hi^ neutrophils from wt and *Il6*^−/−^ mice. Circulating neutrophils were loaded ex vivo with the peroxidase substrate APF and exposed to opsonized *S. epidermidis* labeled with DDAO Far Red. These reporter dyes were used to track neutrophil respiratory burst and phagocytosis capabilities. Circulating Ly6B^hi^Ly6G^hi^ neutrophils from wt and *Il6*^−/−^ mice showed no differences in effector functions (Supplemental Fig. 2F). However, infiltrating neutrophils from *Il6*^−/−^ mice treated i.p. with 5 × 10^8^ CFU of fluorescently labeled *S. epidermidis* displayed impaired neutrophil function (Fig. 3D, Supplemental Fig. 2G). We further confirmed these findings by visualizing fluorescent bacteria in neutrophils using imaging flow cytometry (Supplemental Fig. 2H). Because IL-6 requires sIL-6R to regulate stromal responses within the peritoneal cavity, we conducted an identical experiment in wt mice treated with the IL-6 *trans*-signaling antagonist soluble gp130 (sgp130). Treatment with sgp130 significantly reduced the effector properties of infiltrating neutrophils in infected mice (Fig. 3D). This defect was reversed by reconstituting IL-6 signaling (via i.p. administration of a chimeric IL-6–sIL-6R fusion protein) in *Il6*^−/−^ mice (Fig. 3D, Supplemental Fig. 2H). Thus, IL-6 governs neutrophil responses to local infection.

### Th1 cells alter STAT transcription factor activity

**FIGURE 4.**
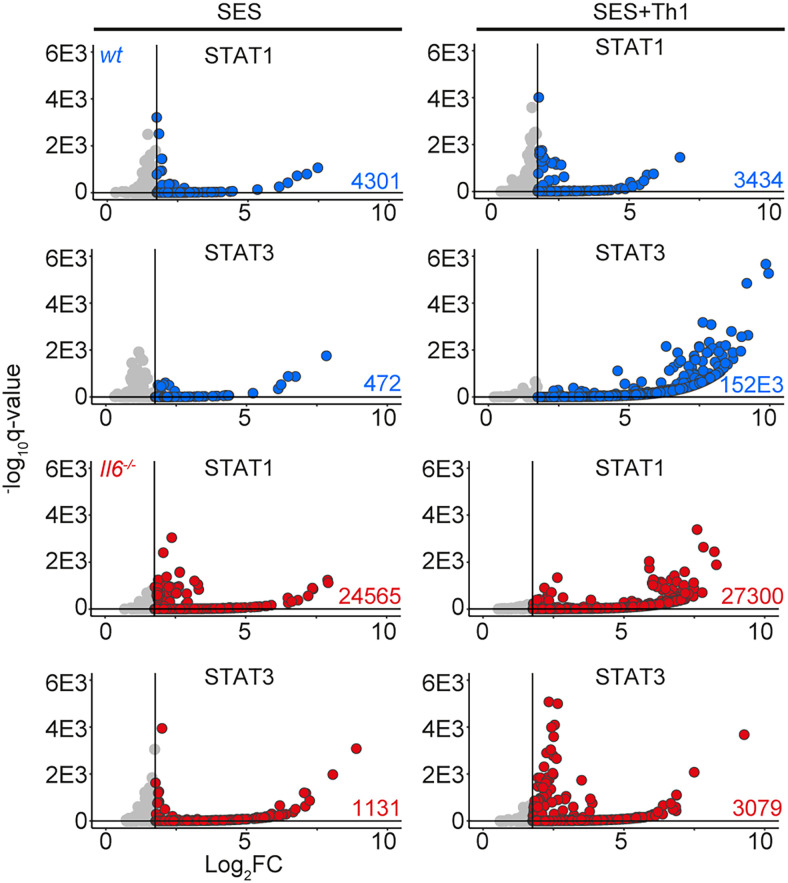
ChIP-seq analysis of STAT1 and STAT3 involvement in SES–induced inflammation. Genomic DNA from the peritoneal membrane of mice challenged with SES alone or SES+Th1 was extracted at 3 h. Peak calling and downstream processing are described in *Materials and Methods*. Volcano plots summarize ChIP-seq profiling. Each dot represents a peak (gray, *q* > 0.05 and/or log_2_FC > 1.75). Peaks below the significance (*q* < 0.05) and above the log_2_FC (>1.75) cutoff values are highlighted in blue (wt) and red (*Il6*^−/−^).

STAT1 and STAT3 transcription factors become rapidly activated following SES challenge, with maximal activation coinciding with the 3 and 6 h chosen for our RNA-seq analysis (22, 35, 38) (Supplemental Fig. 3A). STAT1 activities often shape the transcriptional output of IL-6 and STAT3 (12, 15, 16, 50). To test whether this relationship is seen in response to SES, we extracted peritoneal tissues from *gp130*^Y757F:Y757F^ mice challenged with SES. These animals possess a single tyrosine-to-phenylalanine substitution in the cytoplasmic domain of gp130 that prevents the negative regulation of STAT1 and STAT3 following cytokine activation (35, 40). Immunoblot for pY-STAT1 and pY-STAT3 showed that SES triggers a prolonged STAT transcription factor activation in these mice (Supplemental Fig. 3A). A partial *Stat3* ablation in *gp130*^Y757F:Y757F^ mice (*gp130*^Y757F:Y757F^:*Stat3*^+/−^) extended the duration of pY-STAT1 activity in response to SES challenge (Supplemental Fig. 3A). Thus, STAT1 and STAT3 activities are interlinked in SES inflammation and may explain how Th1 cells impact the transcriptional output of IL-6. We therefore applied ChIP-seq to investigate how STAT1 and STAT3 transcription factors engage the genome following SES challenge (Fig. 4, Supplemental Fig. 3B). Our analysis identified sequencing peaks displaying a 4-fold enrichment above input (*p* < 0.0001; false discovery rate of 0.05). Motif enrichment analysis (MEME-ChIP) confirmed the specificity of these interactions and identified motifs for STAT transcription factors beneath the sequencing peaks (Supplemental Fig. 3C). These mapped to TSS, exons, introns, and intergenic regions (Supplemental Fig. 3D).

**FIGURE 5.**
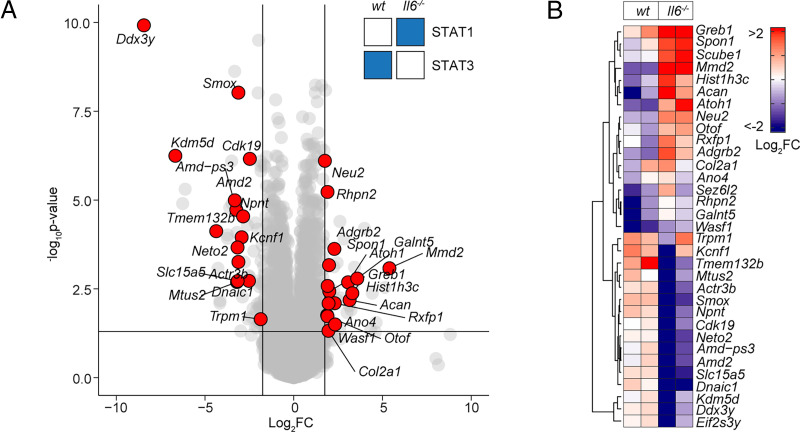
STAT1 and STAT3 interplay shapes gene regulation. (**A**) Volcano plot of RNA-seq data showing differentially regulated gene expression (*Il6*^−/−^ versus wt; *p*_adj_ < 0.05, log_2_FC > 1.75) in SES+Th1–treated *Il6*^−/−^ mice (3 h postadministration). Differential gene regulation is shown for representative genes displaying reciprocal STAT1 and STAT3 binding in ChIP-seq datasets from wt and *Il6*^−/−^ mice (summarized in inset). (**B**) Euclidean clustering of the 33 genes depicted in (A).

The presence of Th1 cells noticeably altered the genomic localization of STAT transcription factors following SES stimulation (Fig. 4). Following treatment with SES alone, STAT1 and STAT3 worked in close partnership and often bound loci in nearby proximity. Th1 cells augmented the number of sequencing peaks identified by ChIP-seq for STAT1 and STAT3, with the bulk of these increases associated with STAT3 binding (Supplemental Fig. 3D). This increase in STAT binding was consistent with the enhanced gene regulation associated with Th1 cell involvement (Figs. 1, 3). Comparing STAT transcription factor binding in SES+Th1 datasets from wt and *Il6*^−/−^ mice, we also identified genomic loci displaying evidence of STAT1 and STAT3 cross-regulation (Fig. 5). STAT transcription factors often bound similar genomic coordinates at downstream sequences distal of TSS. In DNA samples from wt mice, these sites were STAT3 occupied. However, in *Il6* deficiency, these same sites showed increases in STAT1 binding. Promoters displaying this form of cross-regulation included genes involved in cytoskeletal organization (e.g., *Mtus2*, *Actb*, *Rhpn2*, *Wasf1*), metabolism (e.g., *Angptl4*, *Mtor*, *Neu2*, *Pgm1*), and tissue remodeling (e.g., *Timp1*, *Col2a1*, *Vegf*) (Fig. 5A). This switch from STAT3 to STAT1 coincided with transcriptional changes marked by the induction or suppression of gene expression under *Il6* deficiency (Fig. 5B). These included the suppression of genes affecting cellular differentiation (e.g., *Dnaic1*, *Eif2s3y*) and increases in genes linked with matrix protein biosynthesis (e.g., *Acan*, *Npnt*, *Col2a1*). Thus, STAT1 and STAT3 coordinate differential impacts on the control of tissue homeostasis.

**FIGURE 6.**
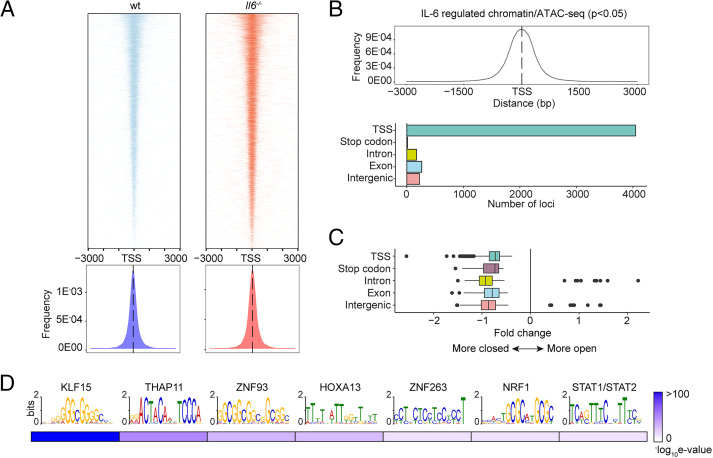
Mapping of chromatin accessibility by ATAC-seq. (**A**) Heatmap visualization of ATAC-seq profiling of peritoneal extracts from mice treated with SES and Th1 cells (top). The peak count frequency of sequence reads associated with transcription start sites (TSS) is shown for wt and *Il6*^−/−^ mice (bottom). (**B**) Histogram shows chromatin accessibility at TSS linked with IL-6–regulated genes (top). Graph shows the genomic distribution of IL-6–regulated loci (bottom). (**C**) Fold change (*Il6*^−/−^ versus wt) in ATAC-seq reads at indicated genomic features. (**D**) Motif enrichment analysis (MEME-ChIP) of genomic regions identified in differential binding analysis of ATAC-seq datasets. Annotations identify putative transcription factor motifs associated with sequencing peaks.

To substantiate the significance of genomic interactions, we used ATAC-seq to confirm links between STAT transcription factor binding and chromatin accessibility in SES+Th1–treated mice (Fig. 6). The heatmap profiles show chromatin accessibility across the entire genome for wt and *Il6*^−/−^ mice (Fig. 6A). Adopting a differential binding analysis (DiffBind) of ATAC-seq datasets, we identified open chromatin regions linked with IL-6 bioactivity. These sites showed enrichment at TSS (Fig. 6B). Access to these sites was partially restricted by the absence of *Il6*, suggesting that IL-6 promotes chromatin remodeling in inflammation (Fig. 6C). Motif enrichment analysis of the DNA sequences aligned to these peaks identified consensus sites consistent with the computational predictions shown in Fig. 2C. These included STAT and IRF transcription factors and others (e.g., KLF15, NRF1, HOXOA13, and several zinc-binding factors) contributing to tissue homeostasis and epigenetic control (Fig. 6D).

### STAT transcription factors engage Alu-like retroelements

**FIGURE 7.**
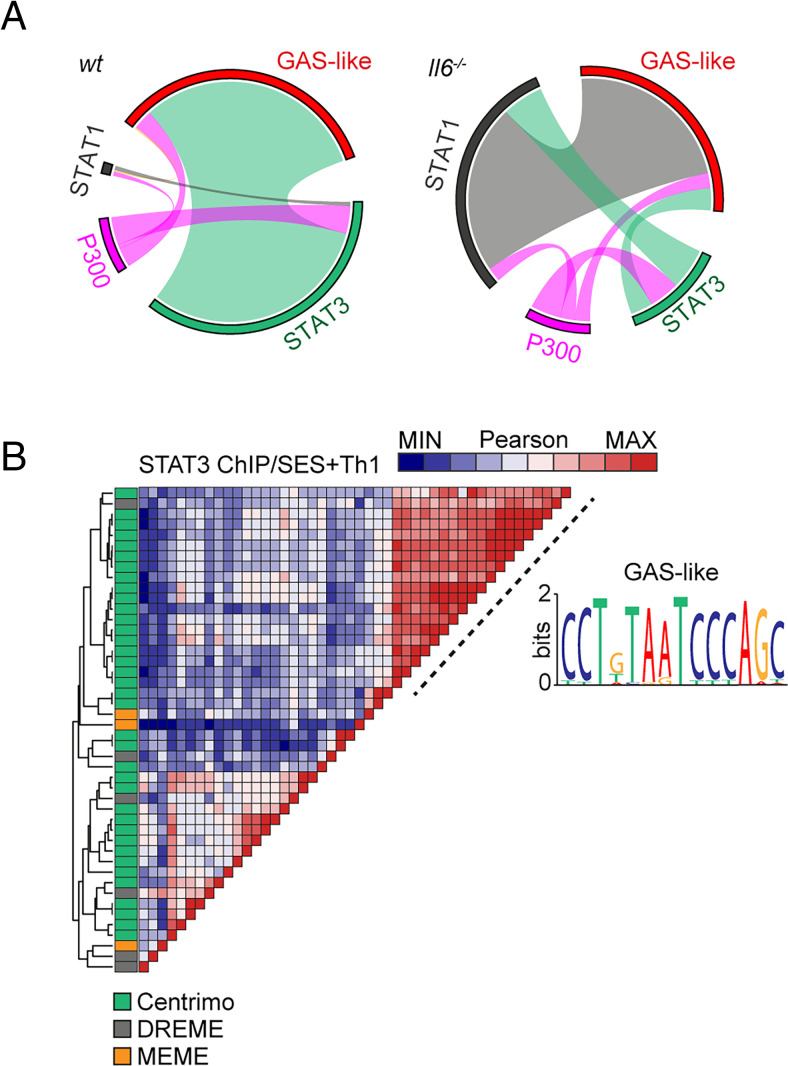
ChIP-seq provides evidence of STAT1–STAT3 cross-regulation. (**A**) Circos plots show the relative binding of STAT transcription factors to putative enhancers defined by either P300 binding (either wt or *Il6*^−/−^ mice) or sites bearing homology to a de novo GAS-like motif identified by STAT1 and STAT3 ChIP-seq (top). Binding to these sites was calculated using the bedtools intersect algorithm. (**B**) Motif enrichment analysis of STAT3 ChIP-seq dataset from wt mice (SES+Th1). Pairwise comparison using the Pearson method was generated using Motif Alignment and Search Tool (MAST; MEME-ChIP suite). Annotation shows the source algorithm of each motif (Centrimo [green], MEME [orange], and DREME [gray]; MEME-ChIP suite). A cluster is highlighted (hashed line) that maps to a sequence displaying homology with a GAS-like motif.

The proximity of STAT1 and STAT3 binding to consensus sequences for other transcription factors suggested links to regulatory regions such as superenhancers. We therefore mapped the genomic localization of P300 in peritoneal tissue extracts from mice challenged with SES+Th1 cells (Fig. 7A). This histone acetyltransferase controls chromatin remodeling and often localizes active or poised enhancers, where P300 functions as a scaffolding factor and coregulator of transcription factor activity (12, 51). ChIP-seq analysis for P300 identified sequencing peaks sharing STAT transcription factor binding (Supplemental Fig. 3B). However, P300 mapping represented a small proportion (<11%) of the total sequencing peaks identified by STAT1 and STAT3 ChIP-seq (Fig. 7A). Although 60% of the P300 loci showed a switch in STAT transcription factor binding under *Il6* deficiency, the transition from STAT3 to STAT1 was more prominent at loci lacking P300 (Fig. 7A). Promoters displaying this motif included genes for *Stat6*, *Adamts1*, *Socs1*, and several IFN-regulated genes (*Irf1*, *Irf9*, *Mx2*, *Il15*, *Ifit1*). These loci displayed STAT3 binding in samples from wt mice and STAT1 binding in datasets from *Il6*^−/−^ mice (Fig. 7A). A limited number of genomic loci showed binding for both STAT1 and STAT3 (Fig. 7A).

**FIGURE 8.**
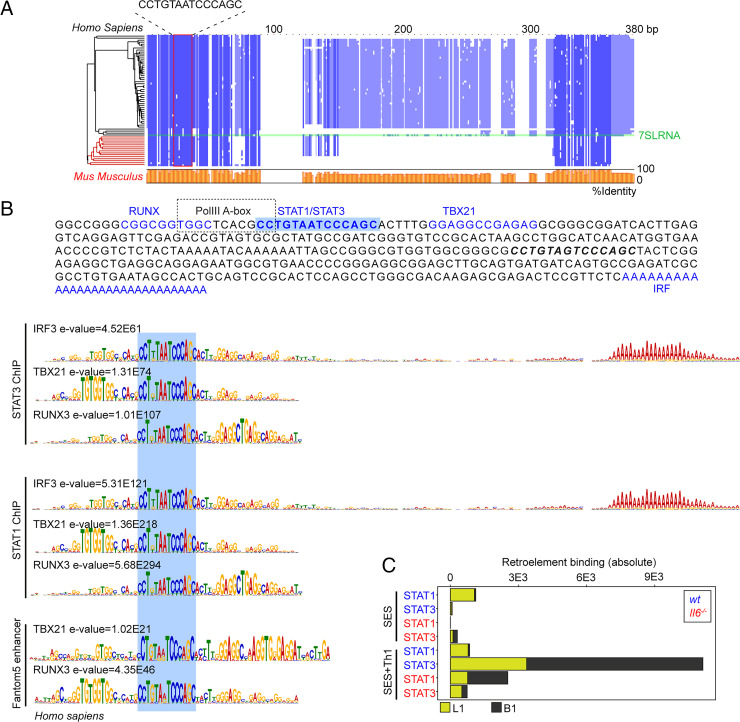
Identification of a GAS-like motif in Alu elements. (**A**) Multiple sequence alignment (MUSCLE; EBI) of human and mouse retroelement sequences downloaded from the Dfam database (*n* = 66 sequences). The Alu-GAS motif (5′-CCTGTAATCCCAGC-3′) identified by STAT1 and STAT3 ChIP-seq is located. Conserved regions are shown in blue, and a summary of the sequence identity is shown in orange (0–100%). (**B**) An annotated summary retroelement sequence is shown (top) locating representative secondary motifs (±150 bp) relative to the Alu-GAS motif. Spaced motif analysis (SpaMo; MEME-ChIP suite) of these secondary sites is shown. (**C**) Quantitation of retroelement binding in ChIP-seq datasets based on RepeatMasker annotations.

Combining wt and *Il6*^−/−^ mice datasets, motif analysis identified a centrally enriched sequence (5′-CCTGTAATCCCAGC-3′) with 90–95% identity to annotated GAS elements (MA0137.3, MA0144.2; Supplemental Fig. 3C) in our STAT1 and STAT3 ChIP-seq data (Fig. 7B). Given the conserved nature of this sequence, we used coordinate mapping to define genomic regions with proximity to the 5′-CCTGTAATCCCAGC-3′ motif in the murine genome. Sequence analysis showed the 5′-CCTGTAATCCCAGC-3′ motif to reside in short interspersed nuclear elements classified by the RepeatMasker bioinformatics tool (Fig. 8) (52). Analysis located STAT1 and STAT3 binding to a subset of short interspersed nuclear elements resembling B1 Alu elements (Fig. 8A, Supplemental Fig. 3E). These elements display conserved architectures that include the 5′-CCTGTAATCCCAGC-3′ sequence (termed the GAS-Alu motif), residing close to an RNA polymerase II A-box and flanked by consensus sites for T-bet and Runx3 (Fig. 8B). Genomic DNA from SES–challenged mice showed no significant interaction of STAT transcription factors with the GAS-Alu motif. Thus, Th1 cells modify Jak–STAT cytokine signaling by redirecting STAT factors to genomic *Alu*-like retroelements.

### The GAS-like motif identifies immune pathways linked to human physiology

**FIGURE 9.**
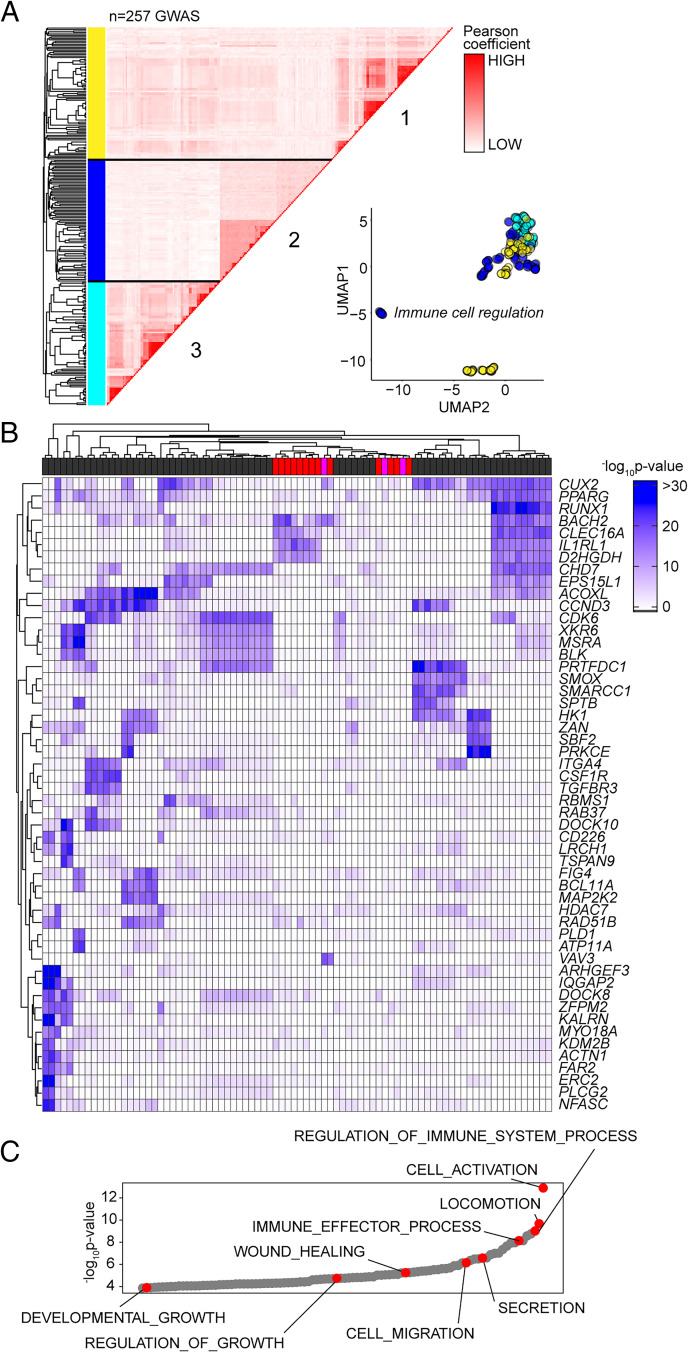
Association of GAS-Alu motif with human physiology. (**A**) Enrichment of GWAS signal in genes containing the GAS-Alu motif. MAGMA output files were compared against enhancers mapped by the FANTOM5 project (see Supplemental Fig. 4). For each GWAS, a vector was formed of the −log *p* values of the motif-linked genes, and Pearson correlations were calculated for each pair of *p* value vectors. Analysis shows the 257 GWAS displaying significant enrichment for associations. UMAP distribution identifies GWAS datasets sharing common traits. Those with links to immune cell function (cluster 2) are highlighted in blue. (**B**) Heatmap of 52 gene targets displaying the top gene-wide *p* values identified in cluster 2 phenotypes. Horizontal bar colors designate GWAS phenotypes linked to immune cell regulation (gray; *n* = 68), immunopathology (red; *n* = 13), and others (pink; *n* = 3). (**C**) Gene Ontology enrichment analysis of the genes identified in (B) (*n* = 53 biological functions). Examples of processes are highlighted in red.

Based on the sequence homology between murine and human Alu elements, we tested whether the GAS-Alu motif correlated with single nucleotide polymorphisms in human disease (Fig. 9A). From publicly available genome-wide association studies (GWAS), we identified GAS-Alu motifs in enhancer sequences designated by the FANTOM5 consortium. Our analysis revealed 8423 sequences, mapping to 2334 genes (Entrez) (Supplemental Fig. 4A). Genes affiliated with the GAS-Alu motif were involved in various processes, including thrombopoietin (e.g., *PRKCB*), VEGF (e.g., *PDGFC*, *ACTG2*), and integrin (e.g., *RAPGEF1*) signaling. Others regulate leukocyte signaling (e.g., *VAV1*, *CACNG3*, *PPP3CB*) and migration (*ARHGAP8*, *ACTG2*), as well as tissue turnover (e.g., *MYO10*, *ARHGEF19*). These included several mapped by our ChIP-seq of murine STAT1 and STAT3; for example, *ARHGEF19* (*Arhgef19*), *COL5A* (*Col5a*), *MYO10* (*Myo10*), *PPARG* (*Pparg*), *PRKCB* (*Prkcb*), and *RABGEF1* (*Rabgef1*).

Next, we downloaded GWAS summary statistics (*n* = 2505) hosted by major repositories (NHGRI-EBI, CTGLab, National Center for Biotechnology Information) and tested for enrichment of GWAS signals in FANTOM5 gene sets and gene signatures of IL-6 and IFN-γ activity (available from MSigDB; Broad Institute) using MAGMA (53). Gene-wide significance levels of genes in these gene sets were compared with a series of randomized and shuffled gene lists. Genes aligned to the HLA locus were excluded from our datasets to control against the high degree of linkage disequilibrium at these loci. For testing enrichment of GWAS signals in genes containing the GAS-Alu motif, MAGMA output files were compared against enhancers mapped by the FANTOM5 project. We identified 257 GWAS displaying enrichment of the GAS-Alu motif (Supplemental Fig. 4B). These included GWAS linked with immune cell regulation, immune pathologies, including asthma, allergy, and heart disease, and others linked with metabolism. A pairwise comparison (Pearson) of genes containing the GAS-Alu motif, based on their GWAS significance, is shown (Fig. 9A). In this study, hierarchical clustering of these GWAS datasets identified three clusters (Fig. 9A). One of these clusters showed enrichment of IL-6 and IFN-γ gene signatures and included genes involved in immune regulation and pathophysiology (Fig. 9B, 9C). These include traits assigned as polygenic and monogenic disease variants (54). Thus, the GAS-Alu motif classifies examples of Jak–STAT cytokine signaling in immune pathology.

## Discussion

Cytokines regulate transcriptional processes that maintain tissue homeostasis, protective immunity, and outcomes affecting inflammation-induced tissue injury (55, 56). In bacterial peritonitis, IL-6 and IFN-γ are critical determinants of these outcomes (4, 10, 22, 27, 31–33, 35, 36, 40, 48, 57–59). These studies concluded that IL-6 compromises tissue repair by supporting the expansion of profibrotic IFN-γ–secreting CD4^+^ T cells, with mice lacking *Il6*, *Ifng*, *Stat1*, or *Rag1* showing resistance to peritoneal fibrosis following recurrent bouts of innate immune activation (22). Although these cytokines rely on STAT1 and STAT3 signaling (22, 35, 38, 40), how these transcription factors coordinate antimicrobial host immunity, tissue scarring, and fibrosis remains unknown. To understand the relationship between IL-6 and IFN-γ in these processes, we tracked the activity of STAT1 and STAT3 transcription factors in stromal tissue following peritonitis. Our analysis shows that IFN-γ–secreting CD4^+^ T cells alter the transcriptional output of IL-6 by channeling STAT transcription factors to previously unoccupied genomic loci.

Jak–STAT signaling is complex and includes an interplay between individual STAT transcription factors (11, 12, 14, 15, 57, 60–63). For example, patients with *STAT1* gain-of-function or *STAT3* loss-of-function mutations often display similar clinical features. These include increased susceptibility to infections at barrier surfaces, eczema-type rashes, and bowel perforations (63–69). These clinical phenotypes are also present in patients lacking *IL6R* or mice lacking *Il6* and often reflect the role of STAT3 in innate immunity (39, 70–75). Consistent with these reports, our analysis shows that IL-6 controls stromal activities that augment the phagocytic properties of infiltrating neutrophils. Th1 cells supplement this response and enhance antimicrobial responses by facilitating increases in IFN-responsive genes supporting host immunity. These data are consistent with the role of IFN-γ in determining neutrophil effector functions relevant to the maintenance of blood pressure, the treatment of chronic granulomatous disease, and innate immune cell involvement in tissue pathology (76–78). During acute resolving inflammation, a limited number of IFN-γ–secreting CD4^+^ T cells are present in the peritoneal cavity (22, 32). This number significantly increases as a response to repeated peritonitis, and their retention leads to compromised tissue homeostasis and fibrosis following activation (22, 32). Our analysis reveals that this change in inflammatory status impacts various cellular processes, including an increase in IL-6 bioavailability and an alteration in IL-6 bioactivity. A previous investigation of acute inflammation in *Ifng*^−/−^ mice highlighted the importance of IFN-γ in steering the IL-6 control of neutrophil trafficking and apoptotic clearance (36). These data point toward a transcriptional interplay between IL-6 and IFN-γ that dictates how STAT transcription factors shape inflammatory outcomes.

Our experimental strategy was designed to understand the relationship between STAT1 and STAT3 signaling in peritoneal inflammation. However, our data also raise questions about how the Th1 cells become activated following an SES challenge of wt or *Il6*^−/−^ mice. Further mechanistic studies are required to address this feature of the model. However, IL-12 remains the most likely orchestrator. The peritoneal cavity is rich in monocytic cell populations responsive to SES. These include F4/80^hi^CD11b^hi^ monocytes and dendritic cell–like F4/80^int^CD11b^int^ populations, which generate IL-12p40 and IL-12p70 following SES treatment (22, 79). This increase in IL-12 is independent of IL-6, meaning that IL-12 could activate Th1 cells under *Il6* deficiency. What is less obvious is whether IL-12 works with other cytokines to optimize the production of IFN-γ. These mechanisms include a potential synergy between IL-12 and the IL-18 receptor system (80, 81). Although we have not assayed changes in IL-18 during peritonitis, our analysis of IL-1β production suggests the rapid activation of the NLRP3 inflammasome system following SES challenge (36).

Studies of cancer cells and ex vivo–stimulated T cells detail how cross-regulation between STAT1 and STAT3 modulates target gene expression (11, 14–16, 23). Our studies suggest that IFN-γ secretion by Th1 cells may facilitate such interactions in stromal tissues following inflammatory activation. In this context, Th1 cells directly impacted the transcriptional output of IL-6 and were responsible for directing STAT1 and STAT3 to previously latent enhancers. Genes affiliated with these loci contribute to tissue remodeling, fibrosis, solute transport, membrane permeability, and hypoxia. Thus, our data points toward an agonist-specific repertoire of latent enhancers employed to sense and interpret changes in the tissue microenvironment (82). For example, transcription factors linked with myeloid cell development (e.g., PU.1) often instruct the binding of NF-κB, AP-1, and IFN response factors to the genome (83). Our results are, therefore, consistent with theories that genes linked by roles in related biological functions commonly share similar mechanisms of transcriptional control (84). In this regard, a close inspection of the DNA sequences enriched for STAT transcription factor binding identified a conserved motif in *Alu*-like retroelements (85, 86). Retroelements are endogenous components of eukaryotic genomes. They support nonallelic recombination, polyadenylation, alternative splicing, and the transcription of gene-rich regions (86, 87). Significantly, retroelements possess consensus binding motifs for various transcription factors and often display evidence of DNA methylation, suggesting an involvement in gene regulation (88, 89). Functional genomic studies in cell lines of stromal or immune cell origin demonstrate the binding of basic leucine zipper transcription factors, the aryl hydrocarbon receptor, and other transcriptional regulators to retroelements (90–92). Our analysis showed that IL-6 signaling, in association with IFN-γ–secreting Th1 cells, promotes STAT3 binding to *Alu* sequences. STAT3 binding was, however, lost in *Il6* deficiency. Instead, these same sites showed STAT1 binding. Thus, *Alu*-like retroelements may represent sentinels of transcriptional cross-regulation in stromal tissues.

What is the significance of STAT transcription factor binding to *Alu* sequences? Are these interactions relevant to human disease or treatment responses to tocilizumab, tofacitinib, and others? Addressing these questions is challenging due to the complexities of mapping repetitive DNA elements. Our data imply a link between *Alu*-like sequences and tissue pathology. Moreover, GWAS commonly identify human *Alu* polymorphisms linked with IFNopathies or diseases characterized by alterations in STAT1 activity (93–95). Our analysis of human GWAS datasets revealed several gene targets also identified in mice treated with SES and Th1 cells. These data support a role for epigenetic modifiers that regulate the accessibility of transcription factors to specific enhancers under certain inflammatory settings or disease processes. Future evaluation of these events will open new opportunities to understand how cytokine cues are interpreted or fine-tuned to direct physiology or pathophysiology.

## Supplementary Material

Supplemental 1 (PDF)
