# Supplementary material for: Th1 cells alter the inflammatory signature of IL-6 by channeling STAT transcription factors to Alu-like retroelements
Source: J Immunol. Author manuscript; Available in PMC 2023 Jul 15. (PMC10315439; doi:10.4049/jimmunol.2300114)
Supplement: Supplementary Figures [file EMS175401-supplement-Supplementary_Figures.pdf]

Supplementary Figures & Legends

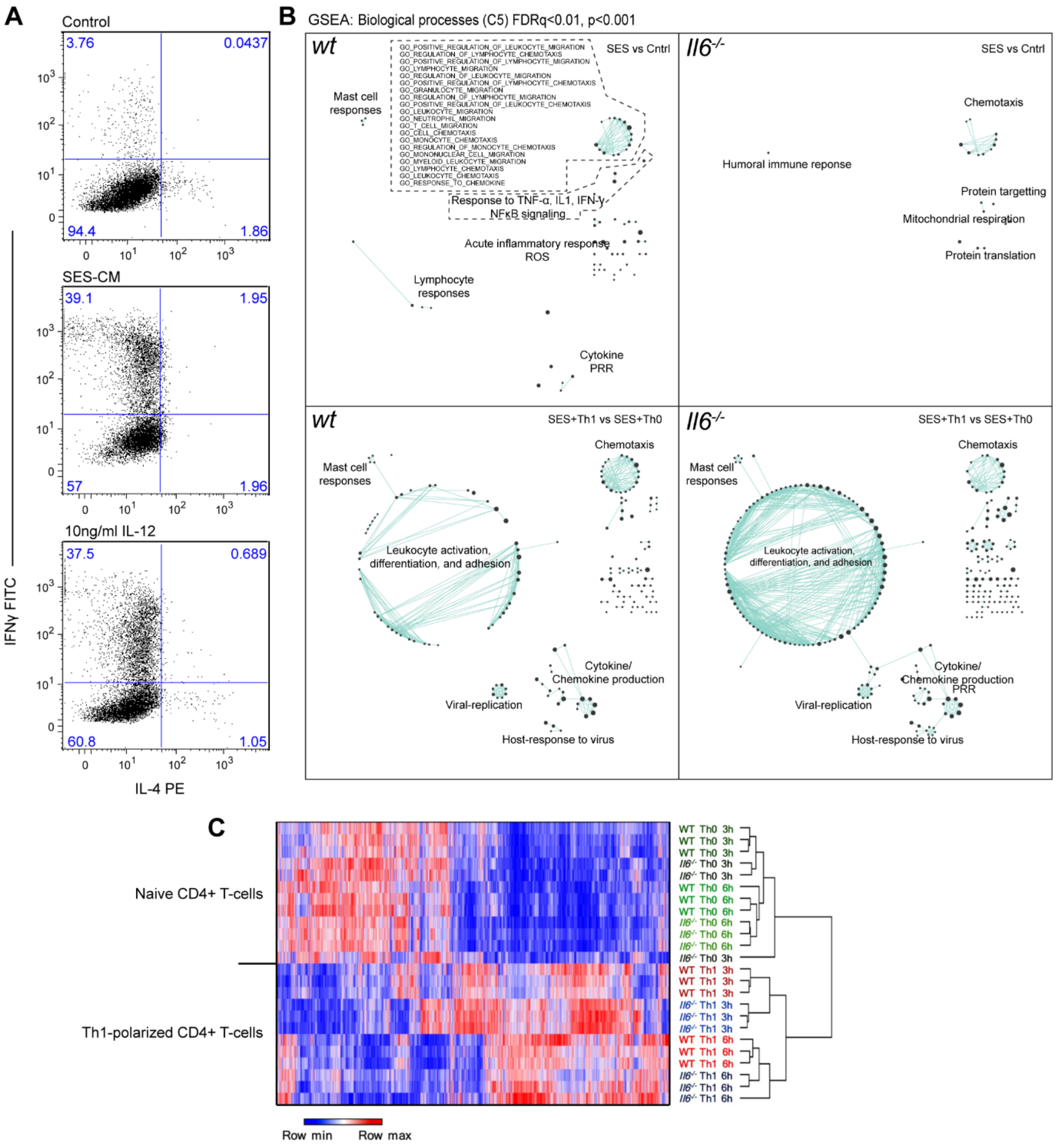

Supplemental Figure-1.

The impact of Th1 cells on stromal tissue responses following SES challenge.

(A) Flow cytometry of intracellular cytokine production in CD4$^+$ T-cells. Representative scatter plots are shown for CD4$^+$ T-cells and activated CD4$^+$ T-cells cultured in the presence of IL-12 or conditioned medium from SES treated peritoneal monocytic cells. Cells derived in this fashion were used in the SES transfer model. Additional information on the expansion of these cells is provided elsewhere (22). **(B)** Enrichment map (Gary Bader; University of Toronto) visualization of Gene Set Enrichment Analysis (GSEA). GSEA was performed for each dataset using a ranked gene list (Log$_2$FC) against the molecular signatures database (msigdb) biological processes (C5; FDRq<0.01, *p*<0.001) reference set. **(C)** Transcriptomic analysis of stromal tissue extracts from SES challenged *wt* and *Il6*$^{-/-}$ mice. Clustering is shown for gene changes following administration of equal numbers of naïve CD4$^+$ T-cells or Th1 cells. Relative transcript levels are clustered by the Euclidean method.

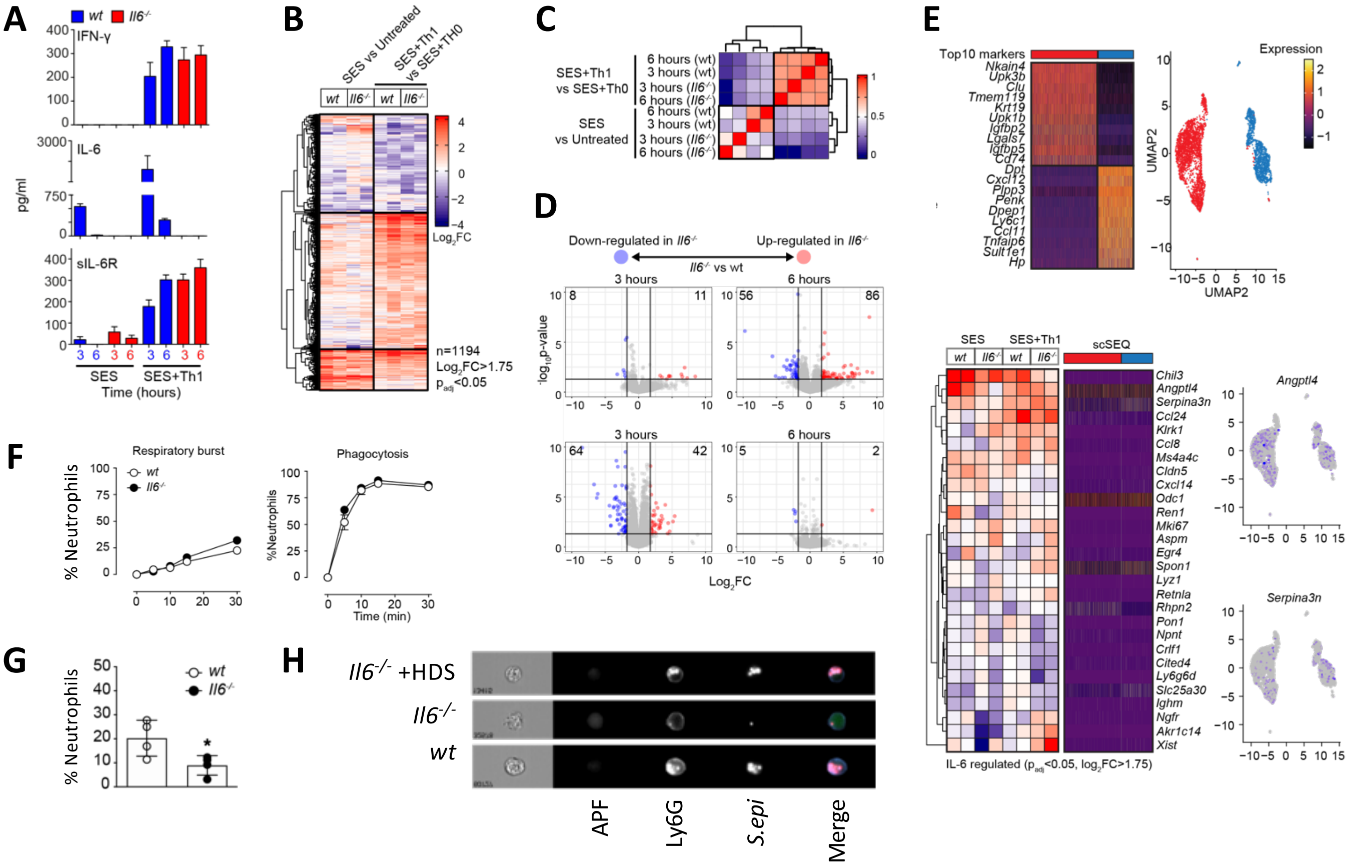

Supplemental Figure 2.

Regulation of gene expression by IL-6

(A) ELISA quantification of cytokines in lavage fluid from SES challenged mice (mean ± SEM; n=4). **(B)** Global transcriptomic analysis as in Figure-1, incorporating data from *Il6*$^{-/-}$ mice. 1194 genes passed statistical thresholds in at least one experimental condition. **(C)** Pairwise comparison of transcriptomic data from Panel-B. **(D)** Volcano plots showing differential expression analysis (LIMMA) of datasets from wt and *Il6*$^{-/-}$ mice (*Il6*$^{-/-}$ vs *wt*). **(E)** Unsupervised clustering of omental stromal cells using available scRNA-seq (GEO: GSM4053741; Ref-49). (Top Panels) UMAP visualization showing classification of fibroblasts (blue) and mesothelial cells (red). The Top-10 genes for each cell type is depicted. (Bottom Panels) heatmap shows a series of IL-6 regulated gene signatures (left) and their corresponding expression in the GEO dataset. UMAP visualizations show two examples (*Angptl4*, *Serpina3n*). **(F)** Flow cytometric analysis of circulating neutrophils from the peripheral blood of *wt* and *Il6*$^{-/-}$ mice. Measurements were recorded over 30-minutes (mean ± SEM; n=3). **(G)** Flow cytometric analysis of infiltrating neutrophils (mean ± SEM; n=4). Neutrophils were recovered from mice were challenged (i.p.) for 6 hours with DDAO-labelled *Staphylococcus epidermidis* (5x10^{8} cfu). **(H)** Corresponding Imaging flow cytometry of infiltrating neutrophils from mice challenged with DDAO-labelled *Staphylococcus epidermidis* (5x10^{8} cfu). Representative images are shown for cells recovered from wt, and *Il6*$^{-/-}$ mice and *Il6*$^{-/-}$ mice treated with an IL-6-sIL-6R fusion protein (50ng/mouse).

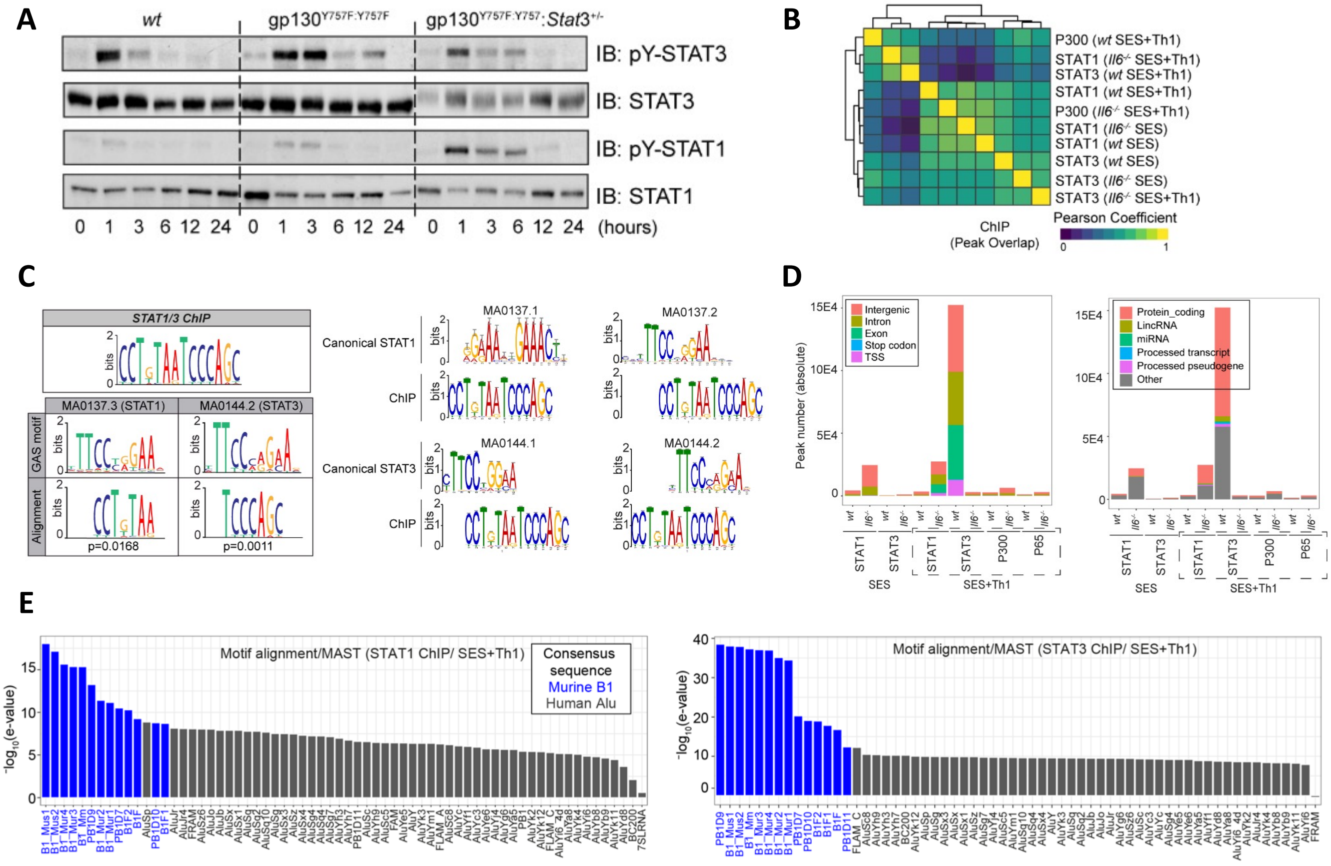

Supplemental Figure 3.

STAT activation and genomic mapping following SES challenge (A) Immunoblot of STAT1 and STAT3 activity in peritoneal tissue extracts from SES challenged *wt*, *gp130*$^{Y757F:Y757F}$, and *gp130*$^{Y75F7:Y757F}$*:Stat3*$^{+/-}$ mice. Temporal changes in tyrosine phosphorylated STAT1 and STAT3 (pY-STAT1, pY-STAT3) are compared against total STAT1 and STAT3. Note, immunoblotting shown for STAT3 in *wt* and *gp130*$^{Y757F:Y757F}$ mice was previously presented in McLoughlin et al (2005) and is used as a control. **(B)** Pairwise comparison of ChIP-seq datasets for P300 and STAT transcription factors in peritoneal extracts obtained from mice treated with SES alone And SES + Th1 cells. **(C)** GAS-like motif enriched in STAT1 and STAT3 ChIP-seq datasets (SES+Th1 condition). Shown are alignments (TomTom; Meme ChIP suite) to canonical GAS motifs (MA137.1; MA0137.2; MA137.3; MA144.1; MA0144.2). Significant alignment ($p<0.05$) can be achieved by breaking down the sequence into constituent parts (top right). **(D)** Histograms showing the number of peaks mapping to genomic features (ChIP-seq). **(E)** Enrichment of B1-like sequences in ChIP-seq datasets. Motif sets generated by Meme analysis of ChIP-seq data were aligned to canonical B1 and Alu sequences downloaded from the Dfam Catalogue (n=66) using the Motif Alignment and Search Tool (MAST; Meme ChIP suite).

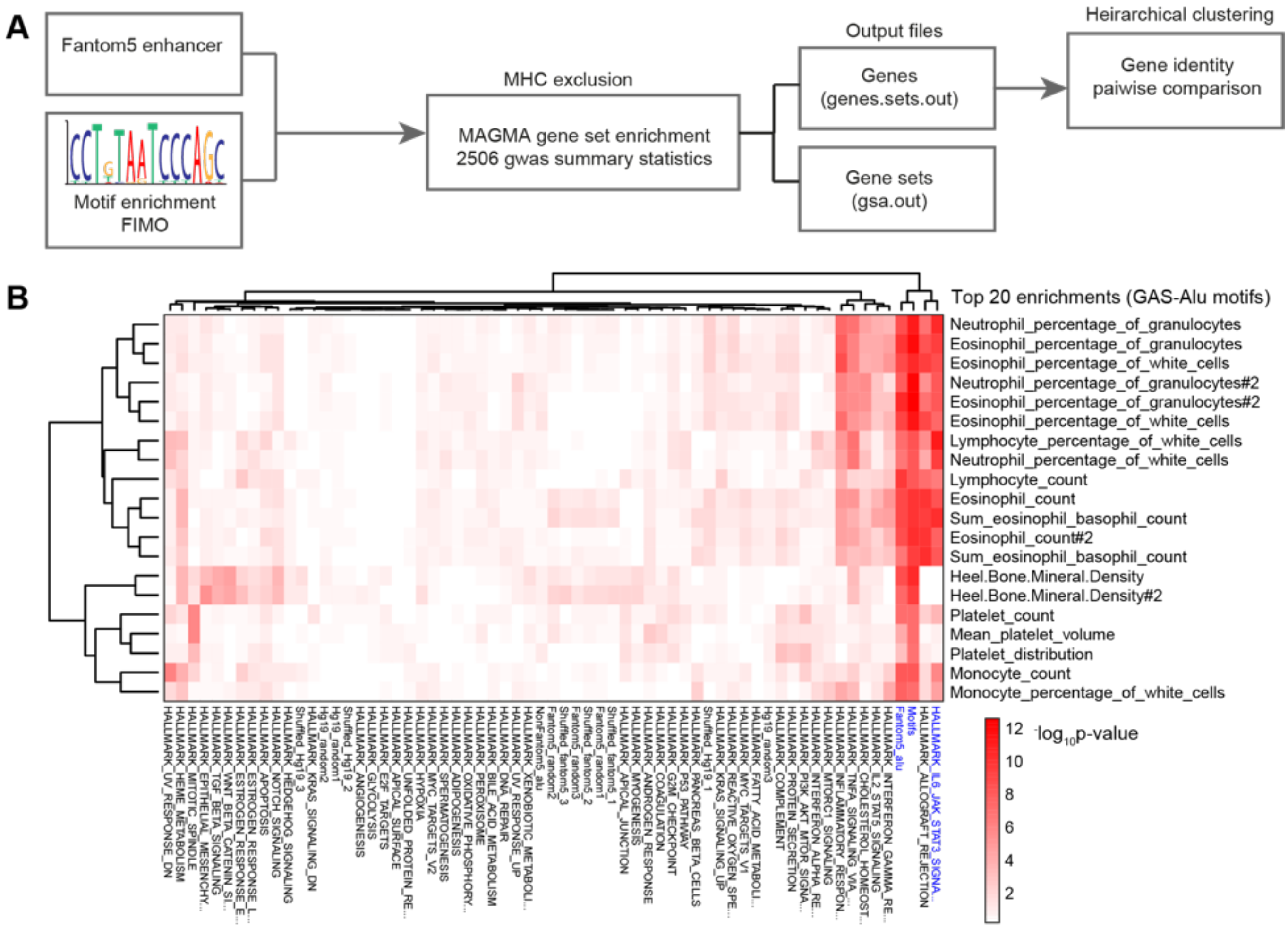

Supplemental Figure 4.

Bioinformatic pipeline underpinning the analysis of GWAS datasets.

(A) Flow chart summarizing the workflow; (1) instances of the GAS-Alu motif were identified across Fantom5 enhancers using FIMO (Meme ChIP suite); (2) Genes linked to these sites were mapped against the Hg19 reference genome; (3) Genes corresponding to the MHC locus were removed prior to MAGMA gene set enrichment analysis against 2506 GWAS summary statistics downloaded from major repositories (EBI, CTGLAB, NCBI); (4) P-values were extracted from MAGMA output files and correlated (Pearson). **(B)** Top 20 enriched phenotypes for the GAS-Alu motif linked gene set. Gene sets were filtered in R so that the highest scoring GAS-Alu motif-linked phenotypes are shown. Heatmap is clustered using the Euclidean method. The x-axis shows gene sets used for the analysis; Hallmark gene sets (msigdb); shuffled and randomized controls; FIMO mapped GAS-Alu motifs in Fantom5 enhancer sequences (motifs); Repeatmasker elements mapping to Fantom5 sequences (Fantom5_alu). HLA-mapped genes were removed prior from all gene sets to MAGMA enrichment analysis.
